# Advances in Cardiovascular Imaging: A Platform to Share Recent Research Findings Through Utilization of Advanced Imaging Technologies

**DOI:** 10.3390/jcdd12100382

**Published:** 2025-09-26

**Authors:** Zhonghua Sun

**Affiliations:** 1 Curtin Medical School, Curtin University, Perth 6845, Australia; z.sun@curtin.edu.au; 2 Curtin Medical Research Institute (Curtin MRI), Curtin University, Perth 6845, Australia

Clinical diagnosis of cardiovascular disease has undergone revolutionary changes over the last decades thanks to rapid developments in medical imaging modalities significantly enhancing the diagnostic performance of cardiac imaging in cardiovascular disease. The diagnostic value of standard cardiac imaging examinations, such as computed tomography (CT), magnetic resonance imaging (MRI), echocardiography, and nuclear medicine technologies including positron emission tomography (PET) and PET-CT, has been augmented by recent advancements, which include the increasing use of three-dimensional (3D) visualizations, namely 3D-printed models, and virtual reality (VR) and mixed reality (MR). Furthermore, the widespread use of artificial intelligence (AI), machine learning (ML), and deep learning (DL), as well as CT-derived fractional flow reserve (FFR-CT), has further expanded the applications of cardiac imaging modalities by delivering more precise or personalized medicine and providing both anatomical and physiological assessment of cardiovascular disease, primarily helping to improve diagnostic assessment of coronary artery lesions. Hence, this Special Issue has been developed to showcase high-quality articles written by researchers from different regions and share the latest research findings in cardiovascular imaging.

This Special Issue includes nine published articles, and though these articles span different study designs, they are all based on the authors’ experiences of using a range of technologies in cardiovascular disease. Four of them are original studies documenting the use of cardiac CT, MRI, PET/CT and 3D visualizations/methodologies in the diagnosis and prediction of cardiovascular diseases [1,2,3,4]. Of the remaining five articles, three are review articles providing an overview of the clinical applications of various imaging modalities in the diagnosis of acute pulmonary embolism [5], chronic total coronary occlusions [6], and FFR-CT and its applications in coronary artery disease [7]. Another review article is a systematic review of AI applications in cardiac MRI in coronary artery disease [8], and the final article is a communication reporting authors’ experience of using mixed reality in guiding cardiac surgery [9]. The following sections summarize key findings from the articles published in this Special Issue.

Nappi et al. reported their experience of using whole-body PET-CT imaging in 109 oncology patients to assess the correlation between coronary artery calcium (CAC) and epicardial adipose tissue (EAT) in these patients without known coronary artery disease (CAD) [1]. Total CAC scores were estimated for three main coronary arteries, while EAT volume was measured and quantified through image processing from the level of the pulmonary trunk to the inferior diaphragmatic surface of the heart by manually tracing pericardial borders. Their results showed that the EAT volume was significantly higher in patients with CAC than in those without CAC (*p* < 0.005). Patient’s age, body mass index (BMI), hypertension, and CAC were significantly associated with increasing EAT values (*p* < 0.005). Based on the multivariable analysis model, only age and BMI were found to be independent factors associated with increasing EAT (*p* < 0.001). This study discusses the feasibility and cost effectiveness of evaluating cancer status and atherosclerotic burden in a single imaging examination in oncologic patients with optimization of radiation dose.

Cardiac magnetic resonance (CMR) is a well-established diagnostic tool for evaluation of patients with suspected myocarditis, and CMR-feature tracking (FT) further advances CMR’s diagnostic accuracy by enabling both morphological and function assessment of myocardial strain. Despite the reported impact of atrial and ventricular functions in patients with acute myocarditis (AM), there is a lack of evidence on the clinical value of CMR-FT in patients with preserved ejection fraction (EF). Cau et al. addressed this research gap by determining the impact of combining atrial and ventricular strain functions using CMR in patients with AM [2]. In this retrospective study based on a single center experience, they reviewed 126 patients with AM with preserved EF, including another 52 age- and sex-matched patients as the control group. Univariable and multivariable logistic regression analysis was used to determine the relationship between left atrial (LA) and left ventricular (LV) functions and presence of AM with preserved EF. LA and LV myocardial strain parameters were found to be significantly associated with impaired function between the AM and control groups (*p* < 0.005). Integration of LA and LV strain parameters into the diagnostic approach improved the diagnostic accuracy of CMR, with the model integrating the global longitudinal strain with the conduit strain achieving the highest area under the curve (0.77). This study highlights how diagnostic performance can be improved by using a combined analysis of both atrial and ventricular functions in patients with AM.

Lee et al. compared four visualization modalities—3D-printed heart models, VR, 3D portable document format files (PDFs), and original digital imaging and communications in medicine (DICOM) images—with regard to their clinical value for diagnostic assessment and preoperative planning of congenital heart disease (CHD) [3]. Cardiac CT images of three CHD cases including ventricular septal defect, double outlet right ventricle, and tetralogy of Fallot were selected, and 3D personalized models were generated from these CT images (Figure 1). These four modalities were presented to 17 participants (13 cardiologists, 3 cardiac surgeons, and 1 radiologist) for assessment of the clinical value of each visualization modality. The participants completed a questionnaire which allowed them to rank the usefulness of these modalities in three main areas, namely pre-surgical planning, medical education, and patient communication. The results showed the superiority of VR and 3D-printed models over 3D PDFs and DICOM images in almost all categories that were assessed in the study, with VR being ranked as the best tool for assessment of anatomical locations, understanding the spatial relationships between cardiac structures and assisting pre-surgical planning. Three-dimensionally printed models were ranked as the best tool for medical education and patient communication, while 3D PDFs and DICOM images have limited value in these areas.

Hashemizadehkolowri et al. proposed a 3D method based on measuring the vessel wall thickness with the aim of overcoming the limitations of 2D techniques [4]. They adopted the Laplacian method, which allows for accurate and reproducible measurements of vessel wall thickness compared to conventional 2D approaches. To achieve their research objectives, the authors conducted a series of experiments based on 2D digital phantoms, 2D histology images (from two carotid artery samples), 3D digital phantoms, and black-blood MR images (from a healthy female volunteer) and measured the vessel wall thickness for comparisons. Their results showed the superiority of the Laplacian method over the current 2D-centric approaches in vessel wall measurements, as it provided accurate 3D thickness measurements based on 2D and 3D digital phantoms. In patient’s MR data analysis, close alignment was observed in the 3D vessel thickness measurements between the baseline and follow-up scans, showing a high level of reproducibility with use of their 3D method, the adapted Laplacian method. Furthermore, the 3D thickness measurements derived from using the Laplacian method are consistent with other morphological features, especially the normalized wall volume index.

Of the four review articles included in this Special Issue, three of them are narrative reviews overviewing various imaging modalities in the context of cardiovascular disease diagnosis. Cellina et al. reviewed the latest developments of diagnostic imaging techniques, including interventional radiology in acute pulmonary embolism (APE) [5]. Among the standard imaging modalities, they first reviewed the use of high-pitch CT pulmonary angiography (CTPA) with low radiation dose (1.04 mSv), followed by dual-energy CT with higher diagnostic accuracy when compared to single-energy CT in APE detection. They then reviewed the diagnostic value of photon counting CTPA in APE, with reported accuracy of more than 95% according to recent studies. Furthermore, photon counting CT offers superior spatial and contrast resolution, and with the use of virtual monoenergetic images, its diagnostic accuracy is further enhanced for APE detection. Dynamic digital radiography (DDR) is an emerging method for assessing pulmonary perfusion with low radiation dose (0.2 mSv) without use of contrast medium. However, it is mainly used in chronic thromboembolic disease, and its clinical value in APE is not yet validated. DL-based APE detection on CTPA was also reviewed with sensitivity and specificity over 90% according to several systematic reviews and meta-analyses. Finally, they also reviewed therapeutic strategies for managing patients with APE, including catheter-directed thrombolysis, mechanical thrombectomy, and thrombus aspiration.

Kasaeian et al. reviewed the clinical applications of FFR-CT in coronary artery disease [6]. FFR-CT has been increasingly reported in the literature to yield improved accuracy in guiding patient management compared to standard coronary CT angiography (CTA). Real-world evidence of using FFR-CT in clinical practice has been validated by a number of multicenter trials proving that FFR-CT serves as a gatekeeper to invasive coronary angiography by reducing a significant number of unnecessary invasive procedures (Figure 2 and Figure 3). FFR-CT is associated with significantly lower all-cause mortality, so it can guide confidential clinical decision-making. Judicious use of FFR-CT to guide coronary CTA interpretation was discussed alongside distal-to-lesion FFR-CT measurement, showing improved concordance with invasive FFR, hence further enhancing diagnostic precision. In this study, the limitations of FFR-CT are highlighted, along with the impact of high-quality coronary CTA image acquisition and coronary calcium scores on FFR-CT performance. Future potential applications of FFR-CT, such as its implementation in the evaluation of coronary artery anomalies (Figure 4) and plaque characterization, are also reviewed. These authors also reviewed the role of FFR-CT as a reliable tool to plan percutaneous coronary intervention (PCI) and identify suitable patients undergoing coronary artery bypass grafting. Finally, they summarized the role of FFR-CT combined with risk stratification as a gatekeeper to the catheterization lab to reduce unnecessary invasive procedures without comprising clinical outcomes.

Panuccio et al. provide an overview of current coronary artery imaging modalities for diagnosis of chronic total occlusion (CTO), focusing on CTO-PCI. They discuss the strengths and limitations of each modality considered [7]. Optical coherence tomography (OCT), intravascular ultrasound (IVUS), and coronary CT angiography (CCTA) are reviewed in terms of their clinical value in assessment of CTO lesions. Despite the inherent superior spatial resolution (10–20 µm) allowing for detailed assessment of coronary wall and plaque morphology, OCT is limited by its penetration depth and invasive nature. In contrast, IVUS has several applications in CTO-PCI procedures, as it can be applied in proximal cap ambiguity, support in antegrade dissection and re-entry, support in reverse controlled antegrade and retrograde tracking, and integrated into stent deployment and optimization. IVUS-guided CTO-PCI is associated with higher procedural success rates, significantly lower procedural times, and a significantly lower risk of stent thrombosis. As a non-invasive imaging modality with the advantage of providing 3D visualizations, CCTA provides accurate assessment of plaque features and an overall vessel architecture (Figure 5). Furthermore, CCTA-based scores have shown better predictive performance than ICA-based scores. The authors of this article also discuss the future prospects of CTO interventions, including the use of AI/ML algorithms for automatic image segmentation and plaque quantification and the development of fusion imaging techniques consisting of combining CCTA with real-time fluoroscopy to provide real-time guidance during CTO-PCI procedures.

The final review article is a systematic review of AI applications in CMR for CAD diagnosis [8]. Jimenez-Jara and colleagues searched three databases and identified 106 studies on the use of AI in the analysis of CMR images in patients with CAD. Forty-six studies documented the applications of AI in the classification of CMR images for diagnostic and prognostic purposes in CAD patients. They reviewed the characteristics of these studies by considering several aspects, including classification between healthy volunteers and patients with cardiovascular diseases, risk stratification for major adverse cardiovascular events and patients with arrhythmia-induced mortality, early detection of left ventricular remodeling, identification of normal and infarcted myocardial segments, and other less-studied factors, such as left ventricular paradoxical pulsation. Forty-one studies focused on the segmentation of different cardiac structures, including the myocardium, myocardial infarction scar, myocardial edema, the coronary artery, or adipose tissue, via CMR images. These studies used the Dice similarity coefficient (DSC) to determine AI performance compared to the ground truth, with high performance in AI segmentation of myocardium and infarcted regions being achieved, although there existed large variability in segmentation accuracy due to differences in MR imaging sequences. The remaining 19 studies reported the use of ML algorithms to extract radiomic features from CMR images for the differentiation of CAD patients. Despite showing great potential in improving diagnostic and prognostic accuracy in CAD, further improvements to the radiomics approach combined with AI models are needed before they are implemented into clinical practice.

The last study in this Special Issue is a communication on the application of mixed reality-guided minimally invasive cardiac surgery (MICS). Aye et al. [9] reported their initial experience of using HoloLens^®^ 2 to guide preoperative planning and for real-time intraoperative guidance in three cases requiring MICS, including aortic value replacement via right anterior small thoracotomy (Figure 6), off-pump coronary artery bypass grafting surgery via left anterior small thoracotomy, and pulmonary valve replacement. The case series showed that mixed reality (HoloLens^®^ 2) optimized ergonomics by superimposing the virtual model on the surgical field, and it provided more appropriate access to the operating field, leading to a mean reduction in cardiac surgical procedures by 34.3 ± 3.2 min. Regarding the use of mixed reality in surgery, positive responses were received from the clinical team. Despite the studied benefits of using mixed reality in MICS intraoperative visualization, further studies on a large cohort with inclusion of different cardiac anomalies and more complexities in cardiac disease are warranted.

In summary, this Special Issue showcases researchers’ experiences of utilizing the latest imaging and innovative technologies in the diagnosis of cardiovascular disease. The findings of these nine studies encompass results that could have wide-ranging implications for cardiovascular disease diagnosis, from standard imaging modalities to the latest technologies, including FFR-CT, mixed reality, and artificial intelligence. This Special Issue serves as a useful resource for readers to learn more about the current status and future development of advanced imaging techniques in cardiovascular disease.

## Figures and Tables

**Figure 1:**
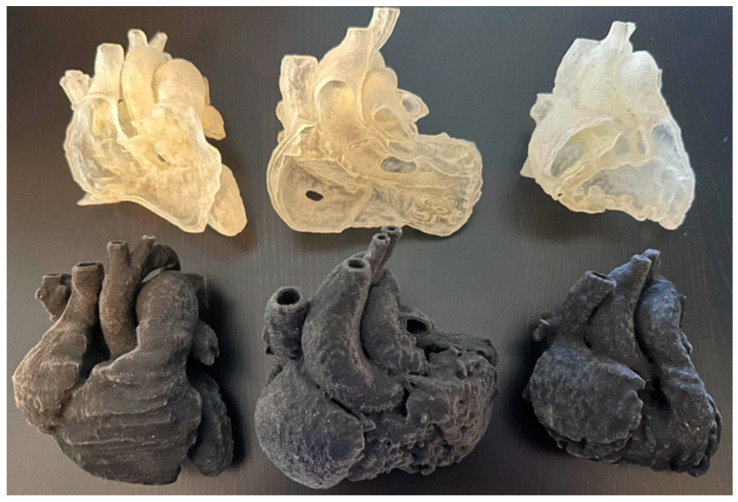
Models were printed with a flexible material, Agilus 30 (Objective 3D, Stratasys, Melbourne, VIC, Australia). VSD (ventricular septal defect) in the left column, DORV (double outlet right ventricle) in the middle column, and ToF (Tetralogy of Fallot) in the right column. Reprinted with permission under open access from Lee et al. [3].

**Figure 2:**
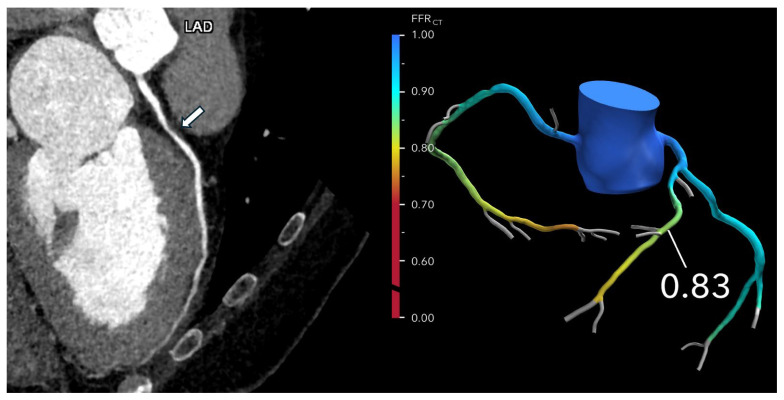
Optimizing treatment decisions with FFR-CT: (**Left**): A curved multiplanar reformatted coronary CTA image of the left anterior descending (LAD) in a 62-year-old man with atypical chest pain reveals severe stenosis (>70%) in the proximal LAD (arrow). (**Right**): The FFR-CT image demonstrates a value of 0.83 measured 2 cm distal to the lesion, indicating normal flow. As a result, invasive coronary angiography was deferred, and the patient was managed with medical therapy. Reprinted with permission under open access from Kasaeian et al. [6].

**Figure 3:**
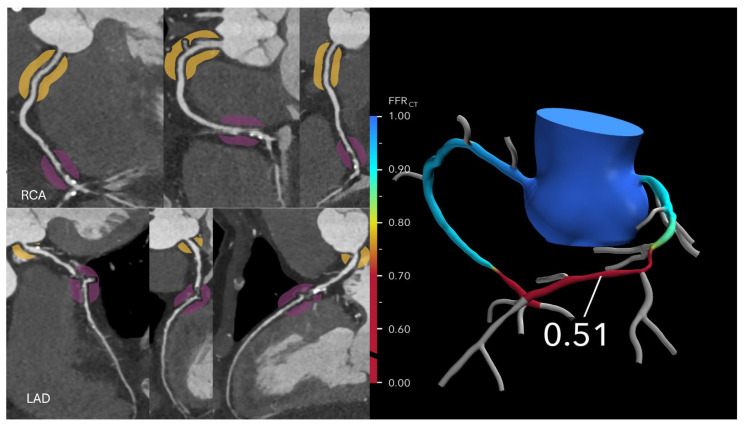
Optimizing treatment decisions with FFR-CT: (**Left**): A curved multiplanar reformatted coronary CTA image in a 64-year-old man with stable chest pain reveals severe stenoses in the mid left anterior descending (LAD) artery and distal right coronary artery. (**Right**): The corresponding FFR-CT image shows a value of 0.51 measured 2 cm distal to the lesion, indicating severely reduced flow. Based on these findings, the patient proceeded to invasive coronary angiography, followed by intervention. Please refer to Figure 4’s caption for the degree of stenosis in relation to different colors. Reprinted with permission under open access from Kasaeian et al. [6].

**Figure 4:**
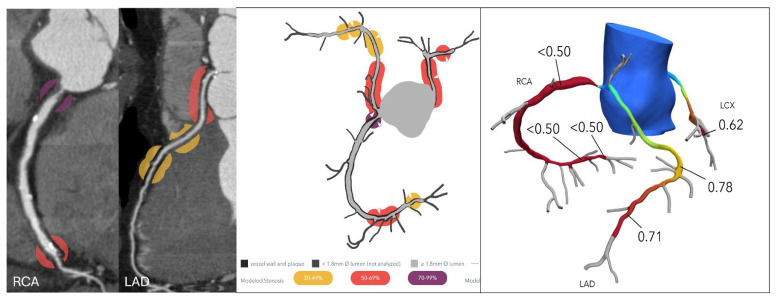
Anomalous aortic origin of the left coronary artery. (**Left**): Curved multiplanar reformatted coronary CT angiography (CTA) images in a 65-year-old male patient demonstrate severe stenosis at the ostium of the right coronary artery (RCA) and moderate stenosis at the ostium of the left anterior descending (LAD) artery, both originating from the right coronary cusp. (**Right**): Corresponding fractional flow reserve computed tomography (FFR-CT) images show a value of 0.50 at 2 cm distal to the RCA ostial lesion, indicating significantly reduced flow. The mid LAD displays a value of 0.78, and the distal LAD shows a value of 0.71, suggesting a high likelihood of hemodynamic significance. Based on these findings, the patient proceeded to invasive coronary angiography and subsequent intervention for both lesions. Reprinted with permission under open access from Kasaeian et al. [6].

**Figure 5:**
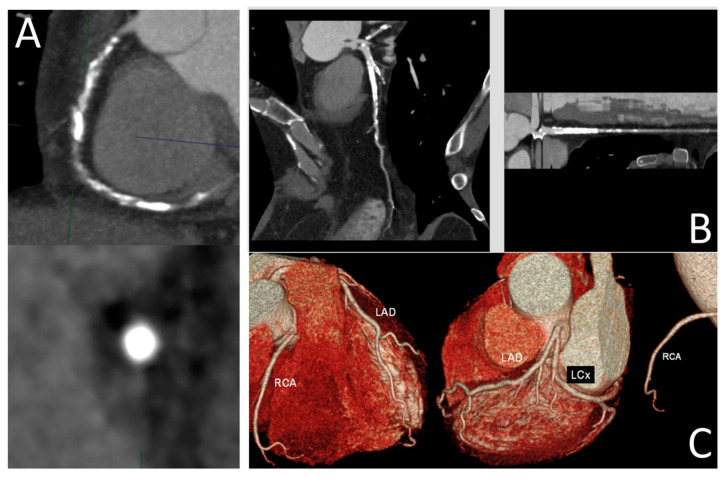
(**A**) Visualization of a high-calcified CTO plaque of a right coronary artery with the slab maximum intensity projection (MIP) technique; (**B**) curved and stretched multiplanar reconstruction of a left anterior descending CTO; (**C**) three-dimensional reconstruction of coronary arteries through the volume rendering approach. Reprinted with permission under open access from Panuccio et al. [7].

**Figure 6:**
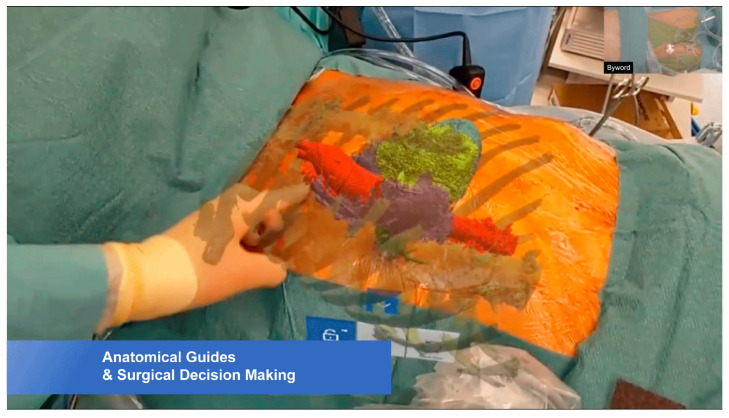
HoloLens^®^ 2-guided minimally invasive surgery for aortic valve replacement. The colors indicate the virtual model overlaying the surgical field. Reprinted with permission under open access from Aye et al. [9].

## References

[1] Nappi C., Ponsiglione A., Vallone C., Lepre R., Basile L., Green R., Cantoni V., Mainolfi C.G., Imbriaco M., Petretta M. (2024). Association of cardiovascular risk factors and coronary calcium burden with epicardial adipose tissue volume obtained from PET-CT imaging in oncologic patients. J. Cardiovasc. Dev. Dis..

[2] Cau R., Pisu F., Muscogiuri G., Suri J.S., Montisci R., Saba L. (2024). Atrial and ventricular involvement in acute myocarditis patients with preserved rejection fraction: A single-centre cardiovascular magnetic resonance study. J. Cardiovasc. Dev. Dis..

[3] Lee S., Squelch A., Sun Z. (2024). Investigation of the clinical value of four visualization modalities for congenital heart disease. J. Cardiovasc. Dev. Dis..

[4] Hashemizadehkolowri S., Akcicel E.Y., Akcicek H., Ma X., Ferguson M., Balu N., Hatsukami T.S., Yuan C. (2024). Efficient and accurate 3D thickness measurement in vessel wall imaging: Overcoming limitations of 2D approaches using the Laplacian method. J. Cardiovasc. Dev. Dis..

[5] Cellina M., Pavan M., Finardi N., Cicchetti F., Ce M., Biondetti P., Lanza S., Carrafiello G. (2025). Advancement in acute pulmonary embolism diagnosis and treatment: A narrative review of emerging imaging techniques and intravascular interventions. J. Cardiovasc. Dev. Dis..

[6] Kasaeian A., Ahmadzade M., Hoffmann T., Ghasemi-Rad M., Ayyappan A.P. (2025). Fractional flow reserve from coronary CT: Evidence, applications and future directions. J. Cardiovasc. Dev. Dis..

[7] Panucio G., Abdelwahed Y.S., Carabetta N., Landmesser U., De Rosa S., Torella D. (2024). The role of coronary imaging in chronic total occlusions: Applications and future possibilities. J. Cardiovasc. Dev. Dis..

[8] Jimenez-Jara C., Salas R., Diaz-Navarro R., Chabert S., Andia M.E., Vega J., Urbina J., Uribe S., Sekine T., Raimondi F. (2025). AI applied to cardiac magnetic resonance for precision medicine in coronary artery disease: A systematic review. J. Cardiovasc. Dev. Dis..

[9] Aye W.M.M., Kiraly L., Kumar S.S., Kasivoishvanaath A., Gao Y., Kofidis T. (2025). Mixed reality (Holography)-guided minimally invasive cardiac surgery-A novel comparative feasibility study. J. Cardiovasc. Dev. Dis..

